# Mitochondria as Inducers of Neutrophil Extracellular Traps

**DOI:** 10.1007/s10753-025-02432-z

**Published:** 2026-01-26

**Authors:** Emil Bečka, Letícia Hudecová, Michal Pastorek

**Affiliations:** Institute of Molecular Biomedicine, Faculty of Medicine, Comenius University, Sasinkova 4, Bratislava, 811 08 Slovakia

**Keywords:** Neutrophil, Neutrophil extracellular traps, Mitochondria, DAMP

## Abstract

Neutrophil extracellular traps (NETs) represent a critical immune defense mechanism that can become pathological in sterile inflammation. Mitochondrial damage-associated molecular patterns (mtDAMPs) emerge as particularly potent triggers of NET formation due to their bacterial-like molecular features inherited from endosymbiotic origins. This review examines the mechanisms by which key mtDAMPs, including mitochondrial DNA, ATP, cardiolipin, cytochrome c, succinate, heme and formylated peptides, induce NETosis through pattern recognition receptors typically reserved for pathogen detection. We describe the complex signaling networks downstream of mtDAMP recognition, highlighting the roles of membrane and intracellular receptors and mitogen-activated protein kinase pathways in orchestrating mtDAMP-induced NET formation. The clinical relevance of mtDAMP-induced NETosis is explored across trauma and wound healing contexts, where neutrophil phenotype along with concentration-dependent and temporal dynamics determine beneficial versus pathological outcomes. Current therapeutic approaches modulating NET formation are discussed challenges in stimulus specificity, pathway redundancy, and use of analgesics and anti-inflammatory drugs. We conclude with future research priorities that include establishing clinically relevant concentration thresholds, elucidating synergistic mtDAMP effects, and developing targeted therapeutic strategies for NET-mediated pathology in sterile inflammatory conditions.

## Introduction

Tissue damage resulting from trauma, ischemia/reperfusion injury, or surgical intervention leads to cell death and release of damage-associated molecular patterns (DAMPs). These endogenous danger signals are detected by tissue-resident dendritic cells and macrophages, which respond by releasing inflammatory mediators that promote vasodilation and increase endothelial permeability [1–3]. Together with other soluble mediators, DAMPs enter the circulation and recruit leukocytes, particularly neutrophils, towards the site of damage. This inflammatory response occurs even in the absence of pathogens, in line with Matzinger’s “danger theory,” which proposes that the immune system primarily responds to threats, rather than merely distinguishing between self and non-self entities [4]. While this process is necessary for proper wound healing, being alerted by damaged tissue can leave the host vulnerable to opportunistic infections elsewhere in the body. Moreover, dysregulation of this response can lead to chronic inflammation and contribute to the pathogenesis of various non-communicable diseases [5–8].

While most DAMPs originate from the cytosol and cell nucleus, mitochondrial DAMPs (mtDAMPs) represent a distinct subset of particular interest [9]. According to the endosymbiotic theory, mitochondria evolved from alphaproteobacteria, and their heritage is reflected by the close similarities between mitochondrial genomes and those of contemporary Rickettsiales [10–12]. Consequently, mtDAMPs possess bacterial-like features, which enable neutrophils to engage mtDAMPs through pattern recognition receptors (PRRs) that typically detect pathogen-associated molecular patterns (PAMPs) [13, 14]. This either primes neutrophils to assist in wound healing through activation of pro-resolving mechanisms (e.g., phagocytosis, VEGF release) or triggers the formation of neutrophil extracellular traps (NETs) [15–17]. Unlike other neutrophil responses, NET formation during sterile inflammation is typically recognized as pathological, perpetuating inflammation, promoting fibrosis, cancer, and causing collateral tissue damage. Interestingly, it was also hypothesized to be beneficial under specific circumstances [18–22].

We therefore aim to highlight that mtDAMP-induced NETosis is a critical but poorly understood mechanism in sterile inflammation. Major knowledge gaps include:

- Information on concentration thresholds that shift neutrophil responses from protective to pathological.
- Identification of the most potent pro-NETotic mtDAMPs amenable to therapeutic targeting.
- Importance of synergistic effects exerted by simultaneously released mtDAMPs that are currently investigated in isolation.
- contribution of microenvironmental factors, host characteristics (age, comorbidities), and trauma treatment modalities that modulate NET formation.

Addressing these questions is particularly important because current therapeutic strategies targeting NETosis remain suboptimal, with pathway redundancy and insufficient clinical translation limiting efficacy. This review therefore systematically examines how mtDAMPs induce NETs during sterile inflammation. Following an introduction to mtDAMPs and NETosis, we detail individual mtDAMP-receptor interactions and their downstream signaling pathways and explore extracellular mitochondrial dynamics. Subsequently, we examine the beneficial versus pathological roles of NETs in wound healing, including modulation by commonly used drugs. Finally, we address NET clearance mechanisms and evaluate current therapeutic strategies.

## Mechanisms of NET Induction

### Lytic NETosis

NETosis was first described by Brinkmann et al., who observed that in order to trap bacteria, neutrophils can expel their chromatin during cell lysis [23]. This lytic NETosis, also known as “suicidal NETosis”, usually takes 1–4 h and can be induced by a variety of microbial and endogenous stimuli, with huge variability reported regarding the concentration of the inducer and time it takes to release NETs [24]. Upon receptor activation by either PAMPs or DAMPs, phospholipase C generates diacylglycerol and promotes intracellular Ca²⁺ release, leading to activation of phosphoinositide 3-kinase (PKC) [25–28]. Multiple PKC isoforms execute dual functions: direct phosphorylation of NADPH oxidase (NOX) subunits (e.g., p47-phox) to initiate reactive oxygen species (ROS) production, and activation of mitogen-activated protein kinase (MAPK) pathways, including the Raf-MEK-ERK and c-Jun N-terminal kinases (JNK) cascades that amplify NOX activity [29–31]. ROS then mediate the dissociation of myeloperoxidase (MPO)-neutrophil elastase (NE) complex localized in azurophilic granules, resulting in the release of NE into the cytoplasm [32]. There, NE degrades actin filaments and translocates to the nucleus to cleave histones, facilitating chromatin decondensation [32]. This is further aided by MPO dimers that disassemble nucleosomes, while monomers produce hypohalous acids with antimicrobial properties [33]. ROS play a central role in lytic NETosis and, apart from initiating MPO-NE dissociation, also promote peptidyl arginine deiminase 4 (PAD4) -mediated histone citrullination, reducing the positive charge on histones and weakening their interaction with DNA [34].

In parallel, apoptosis is suppressed through activation of anti-apoptotic (Akt, Mcl-1) signaling [26, 35]. Cyclin‑dependent kinases (CDK) 4/6 additionally act as important regulatory checkpoints; they are activated independently by cyclin D binding and p21^Cip1^ inhibitor release. Upon translocation to the nucleus, CDK6 enables chromatin modifications during NETosis [36]. NETosis thus involves centrosome segregation and lamins phosphorylation, allowing for an easier DNA release from the nucleus [36–39]. Mechanisms involved in transcription regulation (histone acetylation) also influence NETosis, with dose-dependent effects on NET formation [40]. To finally release the NET into the extracellular space, NE cleaves gasdermin D to its active form. Gasdermin D then creates pores in the plasma and granule membranes, ending in cell lysis [41].

### Non-lytic NETosis

The process of non-lytic, or “vital” NET formation is less explored, but is known to be much faster, with NETs being released within 5–60 min. In non-lytic NETosis, cells release nuclear or mitochondrial DNA (mtDNA), but do not rupture and die. This allows neutrophils to independently perform other functions, such as phagocytosis [42]. Non-lytic NETosis is induced by both exogenous (*S. aureus*, *C. albicans*, lipopolysaccharide (LPS), calcium ionophores) as well as endogenous (activated platelets, GM-CSF, complement) stimuli [43–46].

In non-lytic NETosis, calcium influx through calcium-activated small conductance potassium (SK) 3 channels leads directly to PAD4 activation and histone H3 citrullination. This drives chromatin decondensation and vesicular transport of DNA-protein complexes, allowing for NET release while preserving cell viability [43, 47]. This NOX-independent NETosis was reported to be mediated by SK3 channel and is associated with lower ERK activation and moderate Akt activation, whereas p38 activation is similar to lytic NETosis pathways [35, 47].

**Fig. 1:**
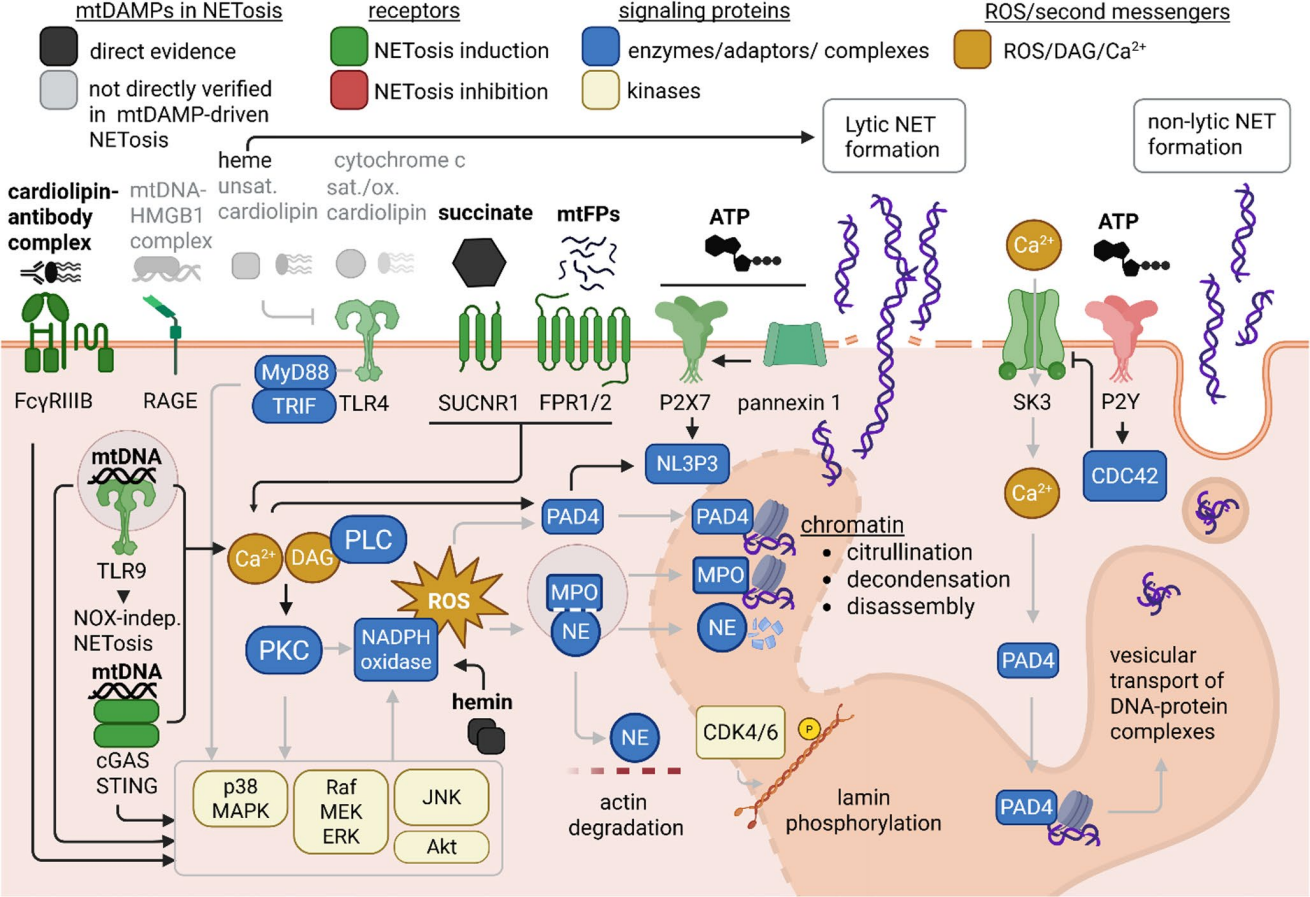
Molecular mechanisms of NETosis and its induction by mtDAMPs. Direct evidence includes verified ligand–receptor interactions and NET release, with key mediators (NOX, MAPK, Ca²⁺) assessed in select cases. Confirmed mtDAMP–receptor pairs: mtDNA (TLR9; cGAS–STING), cardiolipin–antibody complexes (FcγRIIIB), succinate (SUCNR1), formylated peptides (FPR1), and ATP (P2 × 7/pannexin-1). TLR9 and cGAS–STING activation induces p38 MAPK/Akt phosphorylation, contributing to NOX-dependent and NOX-independent NETosis. Hemin triggers ROS-dependent NET formation. ATP–P2 × 7 signaling activates NLRP3 and promotes NETosis but inhibits non-lytic NETosis via P2Y by suppressing SK3-mediated Ca²⁺ influx. Not directly verified as NETosis inducers: cytochrome c and sat./ox. cardiolipin (TLR4 activators), mtDNA–HMGB1 (RAGE binding). Inhibitors: heme and unsat. cardiolipin suppresses TLR4 signaling/expression. Created in BioRender. Pastorek, M. (2025) https://BioRender.com/dk3drp8. Abbreviations: FcγRIIIB, Fc receptor; RAGE, receptor for advanced glycation end products; HMGB1, high‑mobility group box 1; TLR4/9, Toll‑like receptors 4/9; SUCNR1, succinate receptor 1; FPR1, formyl peptide receptor 1; mtFPs, mitochondrial formylated peptides; P2X7/P2Y, purinergic receptors; SK3, calcium‑activated potassium channel 3; MyD88, myeloid differentiation primary response 88; TRIF, TIR‑domain‑containing adapter‑inducing interferon‑β; NLRP3, NOD‑like receptor protein 3; CDC42, cell division control protein 42; NOX, NADPH oxidase; cGAS, cyclic GMP–AMP synthase; STING, stimulator of interferon genes; PKC, protein kinase C; PLC, phospholipase C; DAG, diacylglycerol; p38 MAPK, p38 mitogen‑activated protein kinase; RAF, rapidly accelerated fibrosarcoma; MEK, mitogen‑activated protein kinase/ERK kinase; ERK, extracellular signal‑regulated kinase; JNK, c‑Jun N‑terminal kinase; Akt, protein kinase B; ROS, reactive oxygen species; MPO, myeloperoxidase; NE, neutrophil elastase; PAD4, protein‑arginine deiminase type 4; CDK4/6, cyclin‑dependent kinases 4/6

Alternatively, neutrophils can release just mtDNA. This process takes ~ 15 min and is dependent on mitochondrial ROS [44]. This „mitochondrial NETosis“ is aided by Sirtuin (SIRT) 1, that promotes the opening of mitochondrial permeability transition pore channels or Optic atrophy 1, leading to the reorganization of the microtubule network [48]. Mitochondrial NETosis is particularly interesting in the context of sterile inflammation, as mtDNA itself is a potent DAMP. The signaling leading to both lytic and non-lytic NETosis and its induction by mtDAMPs is illustrated in Fig. 1.

## Mechanisms of mtDAMP-induced NET Formation

NETosis pathways demonstrate significant interconnectedness rather than complete independence. Molecular determinants governing lytic vs. non-lytic NETosis, as well as the extent of true NOX independence, require mechanistic clarification, particularly regarding mtDAMP-induced signaling. Among mtDAMPs, mtDNA, ATP, Succinic acid and heme were described as direct NETosis inducers. mtDNA potently induces lytic NETosis via TLR9/Nuclear factor (NF)-κB and cyclic GMP-AMP synthase (cGAS)–stimulator of interferon genes (STING) pathways without requiring NOX [17, 49]. ATP represents an important modulator of both NOX-dependent and independent NETosis, with sometimes opposing roles [50–55]. Succinic acid induces NETosis through SUCN1 and has been implicated in the exacerbation of uveitis, and its activation predisposes severely injured patients to neutrophil-mediated acute respiratory distress syndrome [56, 57]. Heme has been shown to activate the TLR4/MAPK axis and induce NET formation both in vivo and in vitro [58–60]. While cytochrome c primes the respiratory burst in neutrophil-like HL-60 cells [61].

The role of TLR4 in NETosis during sterile inflammation requires particular clarification. Evidence indicates that mtDAMPs can both activate (cytochrome c, saturated and oxidized cardiolipin) and inhibit (heme, unsaturated cardiolipin) signaling downstream of TLR4. And while TLR4 was implicated in NETosis initiation, its involvement was not extensively studied in case of mtDAMPs [45]. One of the examples is heme, that has been directly linked to suicidal NETosis induction and has also been reported as a TLR4 ligand, but the direct NET formation induction by heme via TLR4 has not been demonstrated [62–65]. Similarly, the receptor for advanced glycation end products (RAGE) has been associated with NETosis and shown to be activated by the mtDNA-high-mobility group box 1 (HMGB1) complex, yet a direct mechanistic link between this specific complex and NET induction remains unconfirmed [66]. Formylated peptides activate neutrophils via formyl peptide receptors (FPR1 and FPR2), leading to phosphorylation of p38 and ERK1/2 MAPK; however, results on their capacity to induce NETs are often conflicting [50, 54, 67].

In addition to signaling that directly leads to NET release, neutrophils also possess mechanisms that inhibit it. Siglec-9 acts as a checkpoint and suppresses NET formation, inducing neutrophil apoptosis [68]. Siglec-5 has also been found to impair NET formation induced by Group B *Streptococcus* [69]. C-type lectin receptors, such as Dectin-1, may prevent NETosis by blocking neutrophil elastase translocation to the nucleus [70]. Myeloid inhibitory C-type lectin-like receptor, an inhibitory C-type lectin receptor, was found to directly recognize DNA in NETs, and a loss or inhibition of its function leads to an uncontrolled auto-inflammatory feedback loop and NET formation [71].

So far, none of these were specifically investigated in the context of mtDAMP-induced NETosis. The following section will provide a detailed overview of particular mtDAMPs and their corresponding receptors and pathways relevant for the induction of NET formation. Impact of particular mtDAMPs on NET formation, along with known pathways and concentration is summarized in Table 1.

**Table 1:** MtDAMPs released during sterile trauma

mtDAMP	mtFP	mtDNA		Heme/hemin	Cardiolipin	ATP		Succinate
**Receptor**	FPR1/2	TLR9	cGAS-STING	TLR4	TLR4; FcγRIIIB; Mac-1	P2 × 7	P2Y2	SUCNR1
**NET formation**	Direct/Priming effect	Direct/Dose-dependent	Context-dependent	Indirect/Dose-dependent	Indirect/Immunocomplexes	Direct	Context-dependent	Context/Dose-dependent
**Molecular pathway**	PKC; NOX-dependent	MAPK; p38; NOX independent	MAPK; NOX; PAD4	ROS; PKC; PRL2	PI3K; Syk; ROS	PKCα; ROS; PAD4	ROS; NOX dependent/independent	PKC; NOX-dependent
**Concentration**	nM-µM	µM	ng/mL-µg/mL	µM	nM-µM	µM-mM	µM-mM	mM
**Reference**	[53, 67]	[17, 49]	[77]	[59, 113, 114]	[101]	[90]	[55, 93, 145]	[57]

*ATP* Adenosine triphosphate, *cGAS*-*STING* cyclic GMP-AMP synthase - stimulator of interferon genes, *FPR1*/*2* formyl peptide receptor 1/2, *Mac*-*1* Macrophage-1 antigen, *MAPK* Mitogen-Activated Protein Kinase, *mtDAMP* Mitochondrial damage-associated molecular patterns, *mtFP* mitochondrial formylated peptides, *NET* neutrophil extracellular trap, *NOX* NADPH oxidase, *PAD4* Peptidyl-arginine deiminase type-4, *PI3K* Phosphoinositide 3-kinase, *PKC* protein kinase C, *PRL2* Phosphatase of Regenerating Liver 2, *ROS* reactive oxygen species, *SUCNR1* Succinate receptor 1, *Syk* Spleen Associated Tyrosine Kinase, *TLR4*/*9* Toll-like receptor 4/9

### MtDNA and TFAM

Phylogenetically related to bacterial DNA, mtDNA contains unmethylated 5’-C-phosphate-G-3’ (CpG) motifs that confer it immunostimulatory properties [14]. In mitochondria, mtDNA is normally stabilized and compacted into nucleoid structures through binding with mitochondrial transcription factor A (TFAM), a dual high-mobility group protein that protects mtDNA from oxidative damage and regulates its transcription. Pathologically, dysfunctional TFAM phosphorylation in conditions like systemic lupus erythematosus can lead to retention of oxidized mtDNA nucleoids within mitochondria and their subsequent extrusion as interferogenic complexes [72]. TFAM dissociation from mtDNA is necessary for normal mtDNA processing and may influence its immunostimulatory potential when released extracellularly, where it was, among other effects, also reported to induce the formation of NETs [14, 17, 73]. mtDNA is primarily recognized through endosomal TLR9, which canonically detects CpG-rich DNA within acidified intracellular compartments and drives the formation of NETs independently of NOX [14, 17]. While TLR9 is known to be expressed on the membrane of intestinal epithelial cells, some studies also suggest its functional presence on the neutrophil surface with a protective role in sepsis, but this phenomenon is likely context-specific rather than constitutive [74, 75].

Notably, purified mtDNA without TFAM protection is highly unstable and exhibits limited capacity to induce NETosis directly, suggesting requirements for co-factors or higher-order complexes [76]. One mechanism enhancing mtDNA recognition involves HMGB1 released during cellular injury. mtDNA-HMGB1 complexes demonstrate synergistic effects on neutrophil activation through concurrent engagement of TLR9 and RAGE, potentially lowering the threshold for response [66]. Additionally, neutrophils can recognize extracellular mtDNA via the cGAS-STING pathway, which parallels TLR9 signaling in activating interferon regulatory factor 3 and NF-κB transcription factors, resulting in type I interferon and pro-inflammatory cytokine (i.e., IL-6) production [77, 78]. In experimental models, both recognition pathways converge on p38 MAPK and PAD4 activation, which are critical for chromatin decondensation during NET formation; however, TLR9 and STING knockout studies show incomplete inhibition of these responses, suggesting involvement of additional pathways [17, 49, 79].

A limitation in the investigation of mtDNA-induced NETosis is the prevalent use of supraphysiological mtDNA concentrations that do not reflect clinical reality. This is further complicated when purified mtDNA is used instead of isolated mitochondria, where mtDNA is more protected against nucleases present in the circulation [76, 80]. The pathophysiological relevance of these mechanisms is nonetheless supported by clinical observations in diverse conditions, including trauma, primary graft dysfunction, cancer, and systemic lupus erythematosus, where elevated levels of circulating mtDNA correlate with NET formation and disease severity [49, 72, 81–83]. NETs themselves incorporate mtDNA alongside nuclear DNA, and some are even predominantly formed by mtDNA, potentially creating a feed-forward inflammatory cycle, more so when oxidized [44, 81, 84]. With aging, neutrophils exhibit heightened sensitivity to mtDAMPs, potentially contributing to inflammaging through enhanced NET-mediated inflammation [85]. Despite these insights, there are gaps present in our understanding of the molecular mechanisms driving mtDNA-mediated NETosis, particularly regarding concentration thresholds, temporal dynamics, and integration with concurrent neutrophil activation cascades.

### ATP

In addition to serving as the primary intracellular energy source, ATP produced by mitochondria can also be actively secreted into the extracellular environment [55]. Once in the extracellular space, ATP exerts a wide range of immunomodulatory effects on immune cells, including the promotion of NET formation [86, 87]. During trauma, damage to both cells and mitochondria leads to the unregulated release of ATP into the extracellular milieu [88]. Neutrophils bind to extracellular ATP via purinergic receptors. The P2Y2 receptor has been implicated in the regulation of neutrophil chemotaxis, whereas members of the P2X receptor family, notably P2 × 7, were shown to be involved in NETosis [89, 90]. Activation of the P2 × 7 receptor triggers downstream signaling pathways that promote assembly of the NOD-like receptor family pyrin domain-containing 3 (NLRP3) inflammasome [91]. This process is further influenced by sterile inflammation, where NLRP3 inflammasome formation is driven in part by the activation of PAD4, leading to histone citrullination and chromatin decondensation [90, 92]. Extracellular ATP may also indirectly activate the small GTPase CDC42 through P2Y receptor, and CDC42 was found to suppress NOX-independent NETosis [93]. Although ATP is generally regarded as a pro-inflammatory mediator, there is currently no clear consensus regarding its overall role in NET formation due to the complexity and concentration-specific nature of ATP signaling pathways.

### Cardiolipin

The inner mitochondrial membrane is rich in various lipids, including phospholipid cardiolipin, which exists in several forms and is tightly packed within the membrane. During cell death, such as in cases of traumatic injury or mitochondrial rupture, cardiolipin can be released from mitochondria [94]. Immunomodulatory effects of cardiolipin are critically dependent on structural configuration and oxidation state. Native unsaturated cardiolipin, the predominant physiological form, acts as a TLR4 antagonist that competitively inhibits LPS-induced inflammation [95]. Pathological conditions promote cardiolipin saturation and oxidation, converting it into a potent TLR4 agonist that mimics bacterial endotoxin and conversely drives NF-κB-mediated pro-inflammatory responses [64]. Additionally, cardiolipin undergoes rapid oxidation during apoptosis through cytochrome c peroxidase activity, representing an early damage signal. That may contribute to sterile inflammation, though its specific role in NET-mediated pathology requires further investigation [96, 97]. The relationship between cardiolipin and NETosis is not yet well defined, with evidence of cardiolipin-driven NET formation appearing mainly in immunocomplex contexts rather than as a standalone stimulus. While cardiolipin can activate the NLRP3 inflammasome, which is associated with gasdermin D-mediated membrane pore formation [98], direct evidence for cardiolipin-induced NET formation is lacking. The strongest connection to NETosis was observed in autoimmune diseases, where cardiolipin and anti-cardiolipin antibodies form immune complexes that activate neutrophils via FcγRIIIB and Mac-1 receptors, leading to NET release [99–102]. The apparent contradictions in the inflammatory effects of cardiolipin reflect the critical importance of distinguishing between its various molecular forms: unsaturated (anti-inflammatory), saturated (pro-inflammatory), and oxidized (highly immunogenic) – as well as the indirect nature of current evidence linking cardiolipin to NETosis through autoimmune rather than direct DAMP signaling pathways.

### Cytochrome C

Mitochondrial membrane permeabilization releases cytochrome C into the cytosol, triggering the intrinsic apoptotic pathway. When released extracellularly, cytochrome C functions as a DAMP, modulating immune responses [62]. While cytochrome C activates astrocytes through TLR4-dependent mechanisms [103] and primes the respiratory burst in differentiated HL-60 cells in response to fMLF [61], direct evidence for cytochrome C-induced NETosis remains elusive [61, 103]. Paradoxically, cytochrome C administration in hemorrhagic shock models reduces oxidative stress, improves lactate clearance, and decreases circulating mtDNA concentration [104].

Nevertheless, cytochrome C may indirectly influence NET-mediated pathology through post-release mechanisms. In systemic lupus erythematosus, cytochrome C can oxidize RNA, presented in complexes with cathelicidin within NETs, potentially enhancing their pro-inflammatory effects on endothelial cells [105, 106]. This observation aligns with findings that oxidized mitochondrial DNA in NETs is interferogenic and contributes to lupus-like pathology [107]. Further supporting this interplay, cytochrome C forms cross-linked complexes with RNA in a hydrogen peroxide concentration-dependent manner [108]. Thus, while cytochrome C may not directly induce NETosis, its extracellular presence potentially enhances the immunogenicity of NETs through its oxidative activity, contributing to autoimmune pathogenesis through post-NET formation modifications.

### Heme

Heme is a vital iron-containing molecule that enables hemoglobin to transport oxygen; in mammals, its synthesis occurs in mitochondria of erythroid cells in the bone marrow and liver [109]. Heme directly binds to the MD-2, an accessory protein of TLR4, activating a robust TLR4-MyD88 downstream signalization pathway, leading to MAPK phosphorylation and NF-κB-mediated cytokine production [110]. Conversely, during severe polytrauma or burns, released heme is associated with impaired immune response and reduced production of proinflammatory cytokines in ex vivo LPS-treated leukocytes, having potential immune “tolerizing” properties [111]. Heme binding on TLR4 leads to downregulation of the TLR4 receptor on the neutrophil surface in an experimental model of liver trauma followed by *S. aureus* lung infection. While neutrophil swarming in the lungs is not affected, bacterial clearance is impaired, suggesting an immunosuppressive effect of heme through TLR4 desensitization [112]. Regarding NETs formation in a sickle cell disease animal model, heme was identified as a direct NET inducer. Furthermore, increased heme concentration correlates with the presence of NETs and can be prevented by depleting heme from plasma [63]. This is in contrast with its reported immunosuppressive properties and requires further clarification.

The oxidized form of heme, hemin, induces NETs formation in a ROS-dependent manner, but with TLR4-independent signaling [113]. In malaria, heme is known to trigger the formation of NETs and the drug hydroxychloroquine can mitigate the resulting tissue damage by preventing the degradation of PRL2 [114]. Meanwhile, hydroxychloroquine also exerts a TLR-9 inhibitory effect, which prevents neutrophil activation in response to released mtDNA [115]. Although it has been shown that extracellular heme induces NET formation, it needs to be elucidated whether its oxidized form alone is enough for the induction or the presence of another mtDAMP, such as mtDNA, is needed.

### Succinate

Unlike classical mtDAMPs, succinate functions as both a metabolic intermediate and a signaling molecule. During cellular stress, elevated succinate acts as a context-dependent DAMP by binding to SUCNR1 on immune and non-immune cells, triggering Ca²⁺ flux and PKC activation [116]. Inflammatory capacity of succinate stems primarily from its ability to stabilize hypoxia-inducible factor-1α, subsequently inducing IL-1β production [117]. In contrast to cytochrome C, succinate directly promotes NETosis. In experimental autoimmune uveitis, succinic acid administration increases the concentration of circulating NETs and in vitro studies confirm the capacity of succinate to induce NETosis [57]. This direct NET induction becomes particularly relevant in conditions with elevated succinate concentrations, including ischemia-reperfusion injury, rheumatoid arthritis, and inflammatory bowel disease [118–120]. The unique metabolic-inflammatory properties of succinate make it an attractive therapeutic target for modulating excessive NETosis in autoimmune and inflammatory diseases.

### MtFP and Proteins

Due to their bacterial evolutionary origin, mitochondrial proteins retain formylated methionine residues from their shared translation machinery with bacteria [9, 121]. When released from the mitochondria, mitochondrial formylated peptides (mtFPs) function as potent neutrophil chemoattractants through the activation of FPRs [54, 122]. Notably, mtFPs can engage both FPR1 and FPR2, binding either with equal affinity or with preferential selectivity for receptor over the other [53, 123, 124]. For example, mtDAMPs released following fractures suppress pulmonary immune responses through both FPR1 and FPR2 signaling pathways [125]. FPR2, discovered later than FPR1, exhibits broader ligand promiscuity [126], and the affinity of mitochondrial peptides for either receptor is influenced by structural characteristics and peptide length [127–129]. This is exemplified by MCT-2, a seven amino acid mitochondrial peptide derived from cytochrome b that preferentially engages FPR2; however, C-terminal cleavage to five amino acids shifts its binding preference toward FPR1 [128, 129]. Importantly, FPR2 activation by mtFPs stimulates the neutrophil proinflammatory response via the ERK pathway and exacerbates ischemia–reperfusion injury independently of FPR1, suggesting that both FPR1 and FPR2 play important and complementary roles in mediating inflammation in response to mtDAMPs [54].

The capacity of mtFP to induce the formation of NETs is characterized by observed clinical correlations and a lack of direct experimental evidence. They demonstrate concentration-dependent modulation of neutrophil responses, with varying potencies that can shift cellular activity from chemotaxis to bactericidal functions, including NETosis [53, 67, 130]. The most extensively studied mtFP, ND6, exhibits functional similarities to bacterial formyl-methionyl-leucyl-phenylalanine (fMLF), including high FPR1 affinity and calcium mobilization capacity, but with unique regulatory properties [53]. ND6 can suppress response to physiological chemoattractants such as CXCL-1 and leukotriene B4, while these mediators do not reciprocally inhibit FPR1 response to ND6 [53]. Mechanistically, mtFPs activate signaling pathways strongly connected to NETosis. FPR1 activation triggers calcium influx, initiating downstream cascades that produce PKC-mediated, NOX-dependent ROS, but can also induce mitochondrial oxidative burst [53, 122, 124, 131]. This pathway mirrors that of known NET inducers such as phorbol-12-myristate-13-acetate and calcium ionophore ionomycin [132].

Supporting this connection, bioinformatics analyses identified *FPR1* as a key gene in NET formation during intracranial aneurysm and acute pancreatitis, with experimental validation in animal models [133, 134]. Furthermore, FPR1 blockade alleviates NET formation in kidney transplant rejection and FPR2 knockout mice show reduced NETs in pathogen-induced lung injury [135, 136]. Despite these mechanistic insights, experimental evidence for direct mtFP-induced NETosis remains inconsistent. While some studies report fMLF-induced NETs at 100 nM-1 µM concentrations [137, 138], others find it only augments NET formation by other stimuli [139]. However, most groups report no NET formation induction by fMLF alone [140–144]. This discrepancy may reflect the complexity of FPR signaling, where FPR1 and 2 display homologous desensitization upon agonist stimulation, which can be nullified upon activation of the ATP receptor P2Y2 [145]. Notably, mTOR inhibition and autophagy induction can permit fMLF-triggered NET formation, suggesting metabolic regulation of this process [144].

Clinically, plasma mtFP levels consistently correlate with NET formation in COVID-19 and systemic sclerosis patients [140, 146]. Paradoxically, mtFPs can also attenuate immune responses through receptor desensitization mechanisms and suppression of responses to other inflammatory mediators, highlighting their dual regulatory role in neutrophil function[147].

## Factors Influencing mtDAMP-induced NETosis

### Presence of Mitochondria in the Extracellular Space

Presence of exogenous and endogenous stimuli often overlaps, with mtDAMPs being released not only during trauma, but also during sepsis or septic shock. Indeed, mtDNA was found as an independent predictor of sepsis mortality and is correlated with disease severity [148–150]. mtDAMPs can additionally affect NETosis through regulating neutrophil availability at the inflammatory site [6, 14, 85, 124]. For example, the CpG-TLR9 axis is responsible for both NETosis and neutrophil extravasation, acting on adhesion molecules expressed on both endothelial cells and neutrophils [151–153]. ATP and mtFP are potent chemoattractants for neutrophils but also prime them for NETosis by increasing intracellular calcium influx [6, 14]. Interestingly, trauma can both pre-activate neutrophils or attenuate them, which is dependent on the surrounding milieu composition as well as temporal aspects [154–157]. However, what part of this phenomenon can be attributed to the increased presence of mtDAMPs is not known. It is therefore crucial to understand not only how mtDAMPs induce NETs, but also their release and stability in the extracellular space.

#### Concentration of Extracellular Mitochondria

Increased concentration of extracellular mitochondria is typically associated with traumatic events, including surgical interventions, where it was found to be predictive of post-operative complications [158]. Beyond acute trauma, aberrant presence of circulating mtDNA is now recognized in various chronic conditions, offering diagnostic and prognostic potential in breast cancer and diabetes [159]. Patients with cardiometabolic disorders also exhibit characteristic mitochondrial signatures, such as elevated extracellular mtDNA, with cellular mtDNA concentration being unaffected [160]. Meta-analyses further confirm moderate positive correlations between circulating extracellular mtDNA, inflammatory markers, and trauma severity [161]. Expanding on these established associations, our recent study of healthy adolescents reveals that circulating extracellular DNA is associated specifically with risk factors for metabolic syndrome rather than with obesity itself [162].

Interestingly, extracellular mitochondria exist in substantial quantities within human circulation even under normal physiological conditions. It was reported that healthy individuals have between 2 × 10^5 and 3.7 × 10^6 intact cell-free mitochondria per milliliter of blood [163]. Others conversely state that while extracellular mtDNA is present in the plasma of healthy subjects, its concentration is nearing the limit of detection [164]. These discrepancies may be likely attributed to a lack of standardization in detection methodologies. Furthermore, platelet activation represents a major confounding factor, as platelets constitute a primary source of extracellular mitochondria [165]. These methodological considerations underscore the need for standardized measurement protocols to evaluate the concentration of circulating mitochondrial components. On the other hand, our findings in adult healthy mice demonstrate considerable natural variability in plasma extracellular DNA (ecDNA) (including mtDNA) and DNase activity levels, even under normal physiological conditions [166]. This suggests that the high variability of extracellular mtDNA may also be partially attributed to a complex relationship between mtDNA release and clearance that we do not yet fully understand.

The presence of mitochondria in circulation under homeostatic conditions suggests physiological roles extending beyond their canonical intracellular functions. Mitochondria can enter the extracellular space in diverse ways: as free organelles, encapsulated within microvesicles, or transferred directly between cells via nanotubes [167]. Activated platelets release functional mitochondria within microparticles, promoting leukocyte activation [168]. Similarly, mtDNA circulates either as cell-free molecules or packaged within extracellular vesicles and exosomes [169]. These likely contribute to their differential immunomodulatory effects. Indeed, mitochondrial transfer from bone marrow-derived stromal cells to pulmonary alveoli demonstrates protective effects against acute lung injury, while mesenchymal stem cells utilize extracellular vesicles to facilitate mitochondrial transfer, influencing macrophage function in clinically relevant models [170–172].

#### Stability of Extracellular Mitochondria

Despite evidence that extracellular mitochondria can trigger NET formation, the quantitative threshold necessary for this effect is not characterized. Our results suggest dose-dependent responses [85]. The synergistic potential of mtDAMPs remains largely unexplored, primarily because experimental approaches typically examine individual components and only a few utilize whole mitochondria/damaged cells. As was previously mentioned, it was suggested that at least three stimuli are needed to induce NETosis [173]. This also aligns with the finding that formylated peptides – despite comprising a significant portion of mitochondrial surface – mostly fail to independently trigger NETs. Similarly, mitochondrially-protected mtDNA demonstrates enhanced stability and immunostimulatory capacity through TLR9 activation compared to naked mtDNA, which rapidly undergoes degradation [174].

Temporal dynamics introduce an additional level of complexity to mtDAMP-neutrophil interactions. Pre-exposing neutrophils to mtDAMPs can, interestingly, suppress subsequent NET generation, a phenomenon observed in trauma patients that can last even 3 days after the initial injury. This inhibitory effect operates through N-formyl peptide-mediated activation of AMPK, which negatively regulates both aerobic glycolysis and NET formation [175]. The stability of extracellular mtDNA further influences its immunomodulatory effects. Oxidative damage promotes double-strand breaks and nucleolytic degradation, with damaged mtDNA typically eliminated within hours [176, 177]. However, oxidatively modified mtDNA exhibits enhanced immunogenicity prior to degradation [178]. On the other hand, Briggs and colleagues reported that following major orthopedic trauma surgery, the number of circulating cell-free mitochondria can remain elevated for five days after surgery [179]. This either means that a portion of mtDNA is protected from degradation, or that the ongoing inflammation promotes continual release of mtDNA.

### Variability in Neutrophil Response

#### Neutrophil Activation

A recent ex vivo study shows that at least three distinct biological triggers are required for effective NET formation [173]. This observation implies that to trigger NETosis, a coordinated activation of multiple receptors is required [70, 173]. Physiologically, DAMPs do not act in isolation, so mechanistic insight from studies using purified molecules may be valuable, but lacking in necessary complexity [180]. mtDAMPs engage a similar set of receptors to PAMPs, but induce NET formation with lower intensity compared to bacterial stimuli [85]. The distinction between a PAMP and an mtDAMP-triggered response is thus very likely dependent on the surrounding environment, rather than specific signaling unique to each. Pro-inflammatory cytokines that are elevated during inflammation enhance the overall neutrophil response, including NETosis. Given that pathologies with a sterile inflammatory component (e.g., stroke, obesity, autoimmune diseases) exhibit distinct cytokine profiles, neutrophil responses to DAMPs are likely to be correspondingly altered [181]. Additionally, alterations in ROS production and calcium influx (both critical components of NETosis) observed in autoimmune disorders also alter the capacity of neutrophils for activation [182, 183].

#### Neutrophil Phenotype

NETosis induction during sterile inflammation is heavily influenced by neutrophil maturation state, age, tissue localization, and, of course, patient comorbidities and disease context. Metabolic dysregulation influences NET-forming capacity, as neutrophils from diabetic patients show elevated PAD4 expression and increased NETosis [184]. Aged tumor-associated neutrophils (CXCR4 + CD62L low) with hypersegmented nuclei preferentially release mtDNA in a SIRT1-dependent vital NET formation [48]. This age-dependent preference for NETosis is further supported by observations that mature blood neutrophils, but not their immature bone marrow counterparts, are primarily responsible for NET release upon IFN-α/γ priming and C5 stimulation [185]. The age of the patient is also a factor, as we have previously shown that the elderly produce more NETs in response to mitochondria than adults. In addition, these NETs are also more oxidized and resistant to DNase I treatment [85].

Beyond age, neutrophils can also be distinguished by their density, with low-density neutrophils (LDNs) being more prone to robust NET release than their high-density counterparts (HDNs) [186]. This is particularly evident in autoimmune conditions, where LDNs from lupus patients overexpress immunostimulatory proteins and alarmins, translating to increased capacity for NET formation [187, 188]. The pathological significance of this phenotype extends also to cancer and heart failure. Increased propensity of expanded LDNs for NETosis was observed in heart failure, but also in the postoperative abdominal cavity of gastric cancer patients, where it facilitated peritoneal recurrence [189, 190]. However, recent work by Hardisty et al. shows that at homeostasis, LDNs have no functional differences from HDNs. This suggests that density alone may be an inadequate discrete functional factor and that the pro-NETotic phenotype of LDNs may be more linked to pathology-specific conditions rather than intrinsic cellular programming [191].

Additionally, specific surface markers may provide further refinement in identifying NET-prone subsets. We previously demonstrated that a small population of α4 integrin–positive (CD49d+) neutrophils shows particular propensity for NET release when exposed to isolated mitochondria [192]. Notably, these CD49d + neutrophils increase in number during sepsis, where the neutrophil phenotypic shift is even more pronounced. Under such severe conditions, emergency granulopoiesis triggers the emergence of either immunosuppressive or pro-inflammatory neutrophil states, further emphasizing the plasticity of neutrophil phenotypes in response to pathological stress [156, 193, 194].

## Impact of mtDAMP-induced NET Formation on Wound Healing

**Fig. 2:**
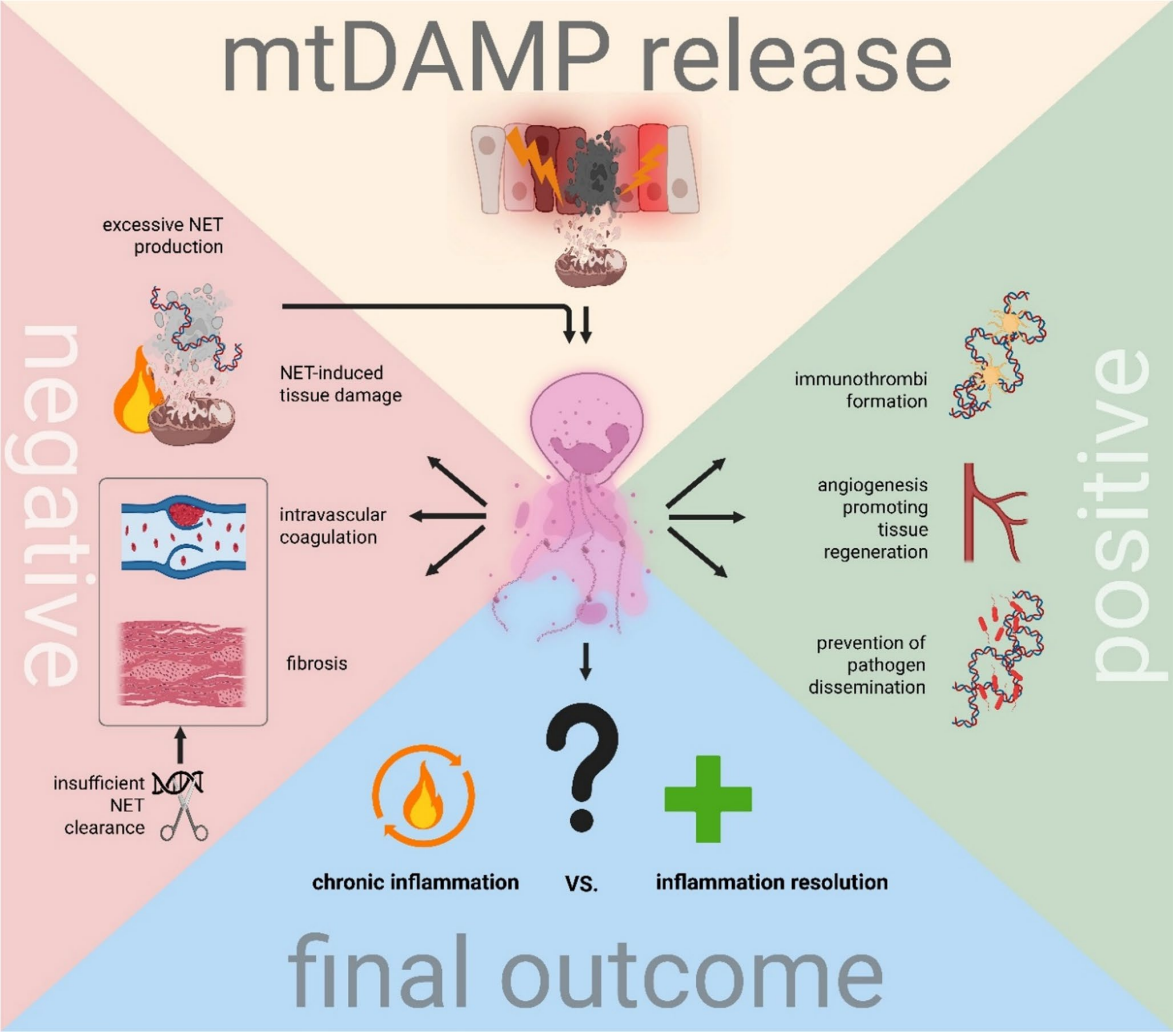
Dual roles of NETs in sterile inflammation. mtDAMP release following tissue injury triggers NET formation. Positive effects: NETs prevent microbial infection, limit pathogen dissemination, promote angiogenesis, and form immunothrombi with platelets to arrest hemorrhage and facilitate tissue regeneration. Negative effects: NET-associated histones and granule proteins are cytotoxic at high concentrations, damaging surrounding tissues and releasing additional DAMPs that perpetuate inflammation and delay wound healing. Excessive NET production drives intravascular coagulation, deep-vein thrombosis, and insufficient NET clearance promotes fibrosis. Final outcome: The balance between chronic inflammation and inflammation resolution depends on context-dependent mechanisms that remain incompletely understood. Created in BioRender. Pastorek, M. (2025) https://BioRender.com/dk3drp8.

In the context of wound healing, NETs have been mostly investigated as drivers of pathology contributing to disease propagation rather than resolution. However, the situation is more complex. NETs released in wounds can prevent microbial infection [195] and blood loss by providing a supportive scaffold for coagulation and thus wound closure [196]. NETs can also be proangiogenic, promoting tissue regeneration before fibrotic tissue develops [197]. Of course, at the same time, properties of NETs that lend them their antimicrobial potential also make them cytotoxic towards host tissue [198]. NET-induced damage to surrounding tissues can therefore result in the release of additional DAMPs and further exacerbation of sterile inflammation [199]. Excessive NETs release that impairs wound healing can also be more pronounced in patients with specific comorbidities, particularly in diabetes [18, 184]. The outcome of NET presence in damaged tissue depends primarily on the wounding context (e.g., surgical vs. traumatic injury, presence of comorbidities such as cancer or diabetes), NET composition, and exposure duration [200]. Both positive and negative impacts of NETs on wound healing are summarized in Fig. 2.

### Positive Impact of NETs on Wound Healing

NET-associated proteases such as NE have cytotoxic potential, cleaving epithelial cadherin in lungs, but simultaneously degrade citrullinated histone H3, one of the most toxic NET components [201]. Cathelicidin-rich NETs may shorten wound healing time by enhancing skin fibroblast migration in vitro [202]. NETs also play a crucial role in coagulation. Following blood vessel damage, both neutrophils and the coagulation cascade are activated to form blood clots and halt hemorrhage [196, 203]. Neutrophils contribute through two distinct mechanisms. First, the NET DNA forms a scaffold for immunothrombi, which aids in stopping bleeding, particularly in damaged intestines, as seen in ulcerative colitis [204]. Supporting the beneficial effects of neutrophil DNA, Ye and colleagues demonstrated enhanced wound healing using DNA-based hydrogels [205]. Second, neutrophil serine proteases such as cathepsin G and NE contribute to the intrinsic coagulation cascade initiation by activating tissue factor and factor XII [206]. NET accumulation at injury sites enriches the area with proteases that can cleave pro-inflammatory cytokines, aiding inflammation resolution [207]. Demonstrating the anti-inflammatory role of NETs, Arya et al. showed in an acute skin inflammation model that swarming neutrophils co-secrete NETs with exosomes containing leukotriene B4. The secreted DNA aggregates these exosomes, concentrating leukotriene B4 in the affected area and helping shift neutrophils from a pro-inflammatory to an anti-inflammatory phenotype, facilitating inflammation resolution [208]. NETs are also associated with proangiogenic activities driven by their pro-inflammatory properties [197]. The role of neutrophils and NETs in wound healing is tightly regulated, and wound healing efficiency in the presence of NETs depends on their composition and circadian rhythm, highlighting new avenues for research and potential applications in regenerative medicine [209]. However, most literature indicates a predominance of adverse effects on wounds and damaged tissue.

### Negative Impact of NETs on Wounds

NET disadvantages in injury and sterile inflammation outweigh their advantages. DNA itself was not reported to be cytotoxic; however, NET-associated proteins, mainly NE and histones, damage surrounding tissues, leading to release of additional DAMPs and further exacerbation of sterile inflammation [199, 210]. Although NET proteins can be cleaved and removed, DNA-protein complexes limit the accessibility of NET-cleaving enzymes, securing NET resistance and prolonging NET-associated enzyme activity [198]. This conformational limitation also affects natural NE inhibition, as inhibitor binding sites within NET complexes become unavailable, leading to extended wound healing times [211]. Excessive NET release damages endothelial and epithelial cells and impairs wound healing, as was already mentioned, particularly in diabetic patients [18, 184].

While regulated NET formation benefits blood clotting, excessive NET release leads to immunothrombus formation, associated with conditions such as deep vein thrombosis and disseminated intravascular coagulation [212]. Large, stabilized NETs form stable clots at injury sites, while smaller NET fragments enhance thrombogenicity [213]. NET production during coagulation is regulated by platelets releasing platelet factor 4, with a phase-dependent role - beneficial for initial hemostasis but potentially problematic for angiogenesis [214]. Tissue repair and regeneration requires endothelial cells, keratinocytes, and fibroblasts to have a coordinated, orchestrated response. NETs at wound sites disrupt this balance by activating Th17 cells, which release IL-17, promoting excessive fibrosis [20].

## The Effect of Pharmacological Interventions on NET Formation in Trauma

Patients with polytrauma are typically admitted to intensive care units, trauma units, or are immediately taken to surgery. Their management and treatment are determined by the severity of their injuries and the therapeutic effectiveness of administered medications. During hospitalization, various analgesic approaches are employed for pain relief, including non-steroidal anti-inflammatory drugs (NSAIDs), acetaminophen, opioids, and local or general anesthesia. In addition, prophylactic antibiotics are routinely administered in trauma patients, particularly those with open fractures, penetrating injuries, or requiring surgical intervention. Understanding how these commonly used medications influence NET formation can have implications for the optimization of trauma care while minimizing potential complications.

### Non-steroidal anti-inflammatory Drugs

NSAIDs are commonly used as first-line treatment for pain and inflammation following traumatic injury [215]. These agents inhibit the production of pro-inflammatory mediators, including chemokines, which leads to reduced neutrophil migration and overall decreased inflammation [216]. While NSAIDs are known to have general immunomodulatory effects, their specific impact on NETosis remains poorly understood. Mechanistically, NSAIDs reduce inflammation by inhibiting cyclooxygenase (COX) enzymes, which block the production of pro-inflammatory metabolites derived from arachidonic acid, such as prostaglandins and thromboxane. This disruption of key biochemical pathways helps decrease both pain and inflammation [217]. Aspirin therapy modulates endothelial cell activity, promoting the synthesis of eicosanoids and the pro-resolving mediator lipoxin A4. While lipoxin A4 increases neutrophil antimicrobial activity through enhancing phagocytic capacity, both aspirin and lipoxin A4 exert inhibitory effects on NET formation and may therefore be beneficial under sterile inflammatory conditions [218–220]. On the other hand, a combination of anti-inflammatory effects might reduce antimicrobial activity during infection [221].

Limited evidence from animal studies suggests NSAIDs may reduce NET formation. Administration of NSAIDs in combination with antiviral drugs in a bovine respiratory syncytial virus infection model showed improved clinical scores and decreased pulmonary NET formation [222].

#### Acetaminophen

Acetaminophen (paracetamol), while not classified as an NSAID due to its minimal anti-inflammatory activity, is frequently used for analgesia in trauma patients. This agent has been shown to reduce neutrophil oxidative burst in hospitalized patients [223]. However, paradoxical effects may occur in overdose situations. Animal models of acetaminophen-induced hepatotoxicity, which can occur with analgesic overdose, demonstrate hepatocyte cytotoxicity leading to acute liver injury. Interestingly, these studies found that AIM2 receptor-dependent NET formation was not observed during acetaminophen toxicity. Instead, mtDNA release induced TLR9-mediated NETosis, suggesting dose-dependent and context-specific effects on NET formation [224, 225].

#### Opioids

Opioid treatment significantly affects neutrophil signaling pathways. Neutrophils recognize opioids through G protein-coupled receptors, leading to downstream calcium influx and can therefore impair neutrophil migration and phagocytosis [226]. A pilot study examining opioid effects on neutrophils found that the synthetic opioid methadone acts as a ligand for both opioid and TLR4 receptors. This dual receptor activation results in increased ROS formation and enhanced NET release, suggesting that opioid therapy may paradoxically promote NETosis in trauma patients [227].

#### Corticosteroids

Critically ill polytrauma patients are often treated with corticosteroids to control systemic inflammation, similar to approaches used in COVID-19-associated acute respiratory distress syndrome [228]. Although direct evidence linking corticosteroid treatment to NET formation remains limited, these agents have been shown to inhibit TLR4-dependent NET formation induced by DAMPs [229, 230]. However, clinical evidence from asthmatic patients suggests that NET concentrations correlate with treatment resistance severity, indicating that corticosteroid therapy may not consistently suppress NETosis across all clinical contexts [231].

#### Anesthetic Agents

Trauma patients typically require surgical intervention under local or general anesthesia. Several ex vivo studies have demonstrated that general intravenous anesthesia can modulate NETosis by inhibiting MAPK pathways and reducing ROS production [232]. In cancer patients, lidocaine administration has been associated with decreased concentrations of NET markers [233]. Similar findings were observed with propofol-epidural anesthesia in patients undergoing colorectal cancer surgery, where this anesthetic combination resulted in decreased serum concentrations of MPO and citrullinated histone H3. Additional ex vivo studies have confirmed reduced NETotic activity in neutrophils treated with lidocaine and bupivacaine [234]. In contrast, dexmedetomidine, used as a supplemental analgesic and anesthetic with potential anti-inflammatory properties, has not demonstrated a significant impact on NET formation, suggesting that not all anesthetic agents equally influence NETosis [235].

Current evidence regarding pharmacological modulation of NET formation in trauma patients by analgesia and anesthesia remains fragmented and largely derived from studies in non-traumatic patient populations with varying disease states and treatment protocols. This limitation significantly hampers direct clinical translation to polytrauma management. Based on available data, anesthetic agents appear to consistently reduce NET formation through multiple inhibitory pathways, including MAPK suppression and ROS reduction. NSAIDs may also decrease NETosis, though evidence is primarily from animal models. Conversely, opioids may promote NET formation through dual receptor activation mechanisms, while corticosteroids show context-dependent effects.

#### Antibiotics

While primarily targeting bacterial infections, emerging evidence suggests that antibiotics can both enhance and inhibit NET formation through direct and indirect mechanisms. β-lactam antibiotics (e.g., imipenem, meropenem, ceftazidime), the most commonly used class in trauma prophylaxis, have been shown to both increase and decrease NET formation by interfering with neutrophil activation pathways, particularly ROS production [236, 237]. Conversely, some aminoglycosides (e.g., gentamicin) may enhance oxidative stress and potentially promote NETosis at therapeutic concentrations [238]. The macrolide azithromycin, increasingly used for its anti-inflammatory properties, suppresses NET formation, but clarithromycin has been shown to induce NETs both in vitro and in vivo [239–241]. Enrofloxacin, a fluoroquinolone antibiotic used in veterinary medicine, enhances the formation of NETs in bovine granulocytes [242]. These findings suggest that antibiotic selection in trauma patients should consider not only antimicrobial spectrum but also potential immunomodulatory effects on NET formation. Furthermore, the effect a particular antibiotic will have on NET formation cannot be inferred from the class it belongs to but must be evaluated independently. Importantly, the timing and duration of antibiotic administration may influence their NET-modulating effects, with prolonged exposure potentially altering neutrophil responsiveness to mtDAMPs.

## NETs Clearance and Therapeutic Targeting

### Mechanism of NETs Clearance

Under physiological conditions, ecDNA in the circulation is primarily degraded by DNase I, which is primarily produced by the liver, kidneys, and endothelial cells. Deficiency or mutations in the DNASE1 gene impair ecDNA degradation, a defect frequently observed in patients with systemic lupus erythematosus [27, 243]. NET degradation involves a more coordinated, multi-step process. DNase1L3, secreted by macrophages, acts on chromatin-associated DNA and enables efficient cleavage of DNA within NET structures [244]. Subsequently, complement proteins opsonize NETs, facilitating their recognition and uptake by macrophages [245]. Within macrophages, NET DNA undergoes further degradation in lysosomes by intracellular exonucleases, including TREX1 and TREX2, completing the clearance [246]. However, it must be noted that this knowledge largely comes from in vitro studies and the efficiency of NET clearance in vivo remains incompletely characterized. In a mouse endotoxemia model, NET DNA was degraded rapidly, yet NET-associated proteins (NE and histones) persisted for several months and induced repeated neutrophil activation [247]. Persistence of NET proteins has important pathophysiological implications, particularly because the NET proteome displays significant variability dependent on the inducing stimuli [248, 249]. Whether and how it differs in response to mtDAMPs has not yet been explored.

Therapeutic strategies employing recombinant nucleases with enhanced activity have been explored to augment physiological NET degradation [250]. However, optimization of administration routes and drug bioavailability remains challenging. This was exemplified in a clinical trial (NCT04355364) evaluating aerosolized dornase alfa (recombinant DNase I) in COVID-19 acute respiratory distress syndrome patients, where successful ex vivo targeting of NETs failed to translate into reduced NET markers in vivo or clinical improvement [251]. These findings highlight the complexity of achieving effective NET degradation in pathological conditions and underscore the need for improved delivery strategies.

### Therapeutical Targeting of NETs

Despite a mechanistic understanding of NETosis, clinical translation remains challenging due to the complexity of selectively targeting pathological NET formation while preserving beneficial neutrophil functions. Current therapeutic approaches can be broadly categorized into four main strategies: upstream mitochondrial protection (using antioxidants like N-acetylcysteine or mitochondrial-targeted compounds) [252–254], receptor-level intervention (TLR9 antagonists, STING inhibitors) [255–257], direct NET formation blockade (PAD4 inhibitors like GSK484) [258, 259], and downstream NET clearance (recombinant DNase therapy) [243, 260, 261]. Successful clinical testing remains challenging. PAD4 inhibitors demonstrate incomplete efficacy in preclinical models and DNase therapy is being predominantly tested in conditions like cystic fibrosis or sepsis. Although promising in animal studies, Gasdermin D inhibition with disulfiram or CXCR2 inhibition of neutrophil activation showed limited efficacy in clinical trials, and there are still no conclusive results for PAD4 inhibitors or nuclease-mediated NET removal available in humans [262, 263]. The answer may lie in the redundancy of NET formation pathways - blocking single mediators often leads to compensatory activation through alternative routes. In trauma patients, where multiple mtDAMPs are simultaneously released, combination therapies targeting different pathway nodes may be more effective than single-agent approaches. Promising emerging strategies should acknowledge temporal context based on injury phase – focusing on protection from mtDAMPs immediately after injury vs. NETosis inhibition and degradation of NETs in its later phases. The possibility of tissue-specific NETosis inhibition could also minimize systemic immunosuppression. Translation success will likely require clinical trial designs that account for the dynamic nature of mtDAMP release and NET formation in real-world clinical settings. Experimental treatments targeting NET formation pathways activated by mtDAMPs are summarized in Table 2.

**Table 2:** Experimental treatments targeting NET formation signaling activated by mtDAMPs

Treatment	Silvelestat	Elamipretide	Coenzyme Q10	Tofacitinib	Digoxin	HCH6-1, Cyclosporin H	MRS2578	Cl-amidine	AS252424	Mdivi-1	Dnase I
**Target**	NE	Cardiolipin	Mitochondrial ROS	JAK	Na+/K+-ATPase pump	FPR1 receptor	P2Y6 receptor	PAD4	PI3Kγ	Mitochondrial fission	NET-DNA
**Approach**	Enzyme-dependent NETosis inhibition	Mitochondrial stability	Mitochondrial stability	Pathway inhibition	Neutrophil calcium metabolism modulation	Receptor inhibition	Receptor inhibition	Enzyme-dependent NETosis inhibition	Pathway inhibition	Mitochondrial stability	NETs clearance
**Advantages**	Direct molecule inhibition	Reduce mtDAMP production	Reduce mtROS production	Specific pathway modulation	Specific pathway modulation	Specific pathway modulation	Specific pathway modulation	Direct molecule inhibition	Specific pathway modulation	Mitochondrial preservation	Remove NETs
Limitation	Incomplete efficiancy	One molecule-specific	Indirect immunosuppresion	Pathway redundancy	Immunosuppresion	Side immunomodulatory effects	Pathway redundancy	Incomplete efficiancy	Pathway redundancy	Non-immunogenic modulation	Late intervention
Development stage	Phase 2/3	Phase 2	Phase 1	Phase 1	Phase 1	Preclinical phase/*in vivo study*	Preclinical phase/ex vivo study	Preclinical phase/in vivo study	Preclinical phase/in vivo study	Preclinical phase/in vivo study	Phase 1/4
**ClinicalTrial.gov ID reference**	NCT05697016	NCT01755858 [264]	NCT02251626	NCT02535689 [265]	NCT03559868	[67, 266]	[267]	[268, 269]	[270]	[271]	NCT05453695, NCT01712334

*FPR1* Formyl peptide receptor 1, *JAK* Janus kinase, *mtDAMP* Mitochondrial damage-associated molecular pattern, *mtROS* mitochondrial reactive oxygen species, *NE* neutrophil elastase, *NETs* neutrophil extracellular traps, *PAD4* Peptidyl-arginine deiminase type-4, *PI3K* Phosphoinositide 3-kinase

## Summary and Conclusion

To advance the field toward clinical translation, several knowledge gaps should be addressed. First, elucidating the hierarchy of mtDAMPs in NETosis is essential to distinguish primary drivers from modulators, while accounting for their synergistic relationships. Current studies examining isolated mtDAMPs do not fully capture the complex milieu following tissue damage, where multiple signals act simultaneously. Second, the development of targeted therapeutic strategies must balance NET inhibition with preservation of antimicrobial immunity. Selective blockade of specific pattern recognition receptors (e.g., TLR9, SUCNR1, FPR1) offers potential for modulating mtDAMP-induced NETosis without compromising broader neutrophil functions. However, the redundancy in activation pathways necessitates combination approaches targeting multiple nodes simultaneously. Third, understanding cell-specific and temporal dynamics of mtDAMP responses is crucial. Neutrophil heterogeneity is influenced by maturation state, density, and metabolic status and it needs to be elucidated how it determines susceptibility to mtDAMP-induced NETosis. Future studies should characterize how patient-specific factors (age, comorbidities, genetic variants) and therapeutic interventions (anesthetics, antibiotics, anti-inflammatory agents) modify these responses. Finally, translational success requires biomarker development to identify patients at risk for excessive NET-mediated pathology and monitor therapeutic efficacy. Real-time assessment of mtDAMP release patterns and NET formation kinetics could guide personalized intervention strategies, optimizing the timing and intensity of treatment based on individual inflammatory trajectories.

## Data Availability

No datasets were generated or analysed during the current study.

## Declarations

### Confirmation of Publication and Licensing Rights for Figures

Figures designed by Hudecová, L. and Pastorek, M. Created in BioRender. Pastorek, M. (2025) https://BioRender.com/dk3drp8.
